# Impact of land use/land cover changes on water quality and human health in district Peshawar Pakistan

**DOI:** 10.1038/s41598-021-96075-3

**Published:** 2021-08-16

**Authors:** Waqas Ahmad, Javed Iqbal, Muhammad Jamal Nasir, Burhan Ahmad, Muhammad Tasleem Khan, Shahid Nawaz Khan, Syed Adnan

**Affiliations:** 1 Institute of Geographical Information Systems, National University of Sciences and Technology, Islamabad, 44000 Pakistan; 2 Department of Geography, University of Peshawar, Peshawar, Pakistan; 3 School of Forest Sciences, University of Eastern Finland, P.O. Box 111, 80101 Joensuu, Finland

**Keywords:** Environmental sciences, Hydrology

## Abstract

The quality and quantity of groundwater resources are affected by landuse/landcover (LULC) dynamics, particularly the increasing urbanization coupled with high household wastewater discharge and decreasing open lands. This study evaluates temporal changes of groundwater quality for 2012 and 2019, its relation to Landuse/landcover, and its impact on Peshawar's residents (study area), Pakistan. A total of 105 and 112 groundwater samples were collected from tube wells in 2012 and 2019. Samples were then analyzed for seven standard water quality parameters (i.e., pH, electric conductivity (EC), turbidity, chloride, calcium, magnesium, and nitrate). Patient data for waterborne diseases were also collected for the years 2012 and 2019 to relate the impact of groundwater quality on human health. Landsat satellite images were classified for the years 2012 and 2019 to observe landuse/landcover dynamics concerning groundwater quality. Results manifested a decrease in groundwater quality for the year 2019 compared to 2012 and were more highlighted in highly populated areas. The nitrate concentration level was found high in the vicinity of agricultural areas due to the excessive use of nitrogenous fertilizers and pesticides, and thus the methemoglobinemia patients ratio increased by 14% (48–62% for the year 2012 and 2019, respectively). Besides, Urinary Tract Infections, Peptic Ulcer, and Dental Caries diseases increased due to the high calcium and magnesium concentration. The overall results indicate that anthropogenic activities were the main driver of Spatio-temporal variability in groundwater quality of the study area. The study could help district health administration understand groundwater quality trends, make appropriate site-specific policies, and formulate future health regulations.

## Introduction

The world's water ecosystem is at high risk due to its changing biological, chemical, and physical properties^1^. Groundwater, which is the primary source for drinking, is deteriorating due to the increasing population growth, urbanization, land use/land cover changes, water demand, and climate change^2^. The combined effects of these changes and nature activities such as droughts and water resources, particularly freshwater, are becoming inadequate and polluted^3,4^. The human population also suffers from water diseases such as methemoglobinemia (blue-baby syndrome) and thyroid effects in bottle-fed infants (less than 6 months old)^5,6^ caused by nitrate polluted portable water. This rapid increase in population also transforms the natural environment into an anthropogenic environment, which means that the anthropogenic activities would take place at a massive scale, such as industrialization and massive food production activities to meet the food and fiber demand the exponential population growth^7,8^. At the dawn of the industrial revolution, humans' life span had decreased due to the consumption of polluted water discharged from the industries and mixed with clean water^9^.

Groundwater is a crucial element that plays a significant role in the health of humans, animals, and aquatic ecosystems. However, this water faces high pollution risks from anthropogenic activities like massive agricultural crop production, urbanization, mining, and industrial developments^10^. Human is the primary cause for the contamination of the groundwater. More agriculture activities will have more chances to contaminate the groundwater because of the excessive applications of pesticides, herbicides, and massive nitrogenous fertilizer applications^11,12^. The impacts of the groundwater contamination are more noticeable in the areas which are more suffering from salinity, desertification, or areas that do not have sufficient groundwater to support intense agricultural activities. An alluvial aquifer is vulnerable to contamination due to several factors, including high permeable alluvial deposits, shallow water table, the interconnection between the agriculture water, and the surface runoff water. Also, direct discharge of domestic wastes, industrial discharge, leakage from the septic tank, and poor farm waste management is the intrusive groundwater contamination source^13,14^.

Pakistan is at a very crucial stage of water scarcity^15^. The water quality level is also below the recommended guidelines of WHO^16^. Pakistan's total annual average groundwater potential is 67,841.4 m^3^, and the abstraction is 51,189.42 m^3^. The water dearth of district Peshawar is 15,141.6472 m^3^, but, in a few decades, it would be 79,493.64779 cubic meters if sustainable management are not adopted. The highest abstraction, which is 81%, takes place in the Punjab province out of which 80% have fresh and clean water while 20% have saline water. Also, Sind province has a 12% abstraction of groundwater in which the freshwater is 23%, and 77% is saline^17^. Khyber Pakhtunkhwa has 5% abstraction of groundwater. As a developing country, Pakistan needs to formulate proper planning about water-related issues such as water availability, quality, and consumption, and protection from diseases resulting from water pollution^18^. The private sector has taken an interest in groundwater development, so the Government of Pakistan assigned boreholes to the private sector^19^. About 6 million residents in Mardan, Peshawar, Nowshera, and Charsada districts of the Khyber Pakhtunkhwa province have no access to clean potable water. The remaining people draw water from dug well and tube wells, which are more susceptible to pollution caused by sewerage lines, toilets, seepage, and percolation of polluted water^20^.

Real-time groundwater monitoring and sampling provide information about the quality and quantity of available water resources, which are important to understand groundwater's current state in the monitored area^8^. Groundwater monitoring and sampling are complex, difficult to apply to a broad area, and very costly compared to groundwater modeling. Groundwater modeling is less complicated and costly and allows us to evaluate broader areas^21^. In-Ground modeling, groundwater samples are collected at random locations, analyzed in the laboratory for various parameters such as temperature, color, hardness, pH, chloride, sulfate, and alkalinity, and their spatial distribution are carried out to detect the aquifer pollution throughout the area which helps to guide efficient groundwater management^22,23^.

Many groundwater studies in China, India, Bangladesh, Iran, and Pakistan have been conducted, and various approaches have been employed to assess groundwater quality^24–27^.

These approaches include regression modeling^26,28^ or modeling based on geospatial data such as geographical information systems (GIS) and Remote sensing (RS)^25,29–32^. These kinds of analyses help identify the intimidations of water quality and provide a vital role for the decision-makers to allow them to take immediate action and ensure groundwater development sustainability^21,33,34^. However, none of these studies, mainly those applied in the Peshawar district (study area) used a holistic approach interlinking the groundwater quality impact on human health and its consequences and formulation of the remedial action plan against the groundwater pollution risk on human health.

The study aimed to exploit the spatio-temporal variation of various groundwater quality parameters and evaluate the effect of landuse/landcover dynamics for the years 2012 and 2019 on the Peshawar district's groundwater quality, Pakistan. Peshawar was selected as our study area due to the rapid changes in groundwater and infrastructure development^35^ and the absence of proper awareness and sustainable management regulations^36^. This study would help to minimize the contamination of groundwater quality by improving the existing water management policies.

## Materials and methods

### Study area

**Figure 1:**
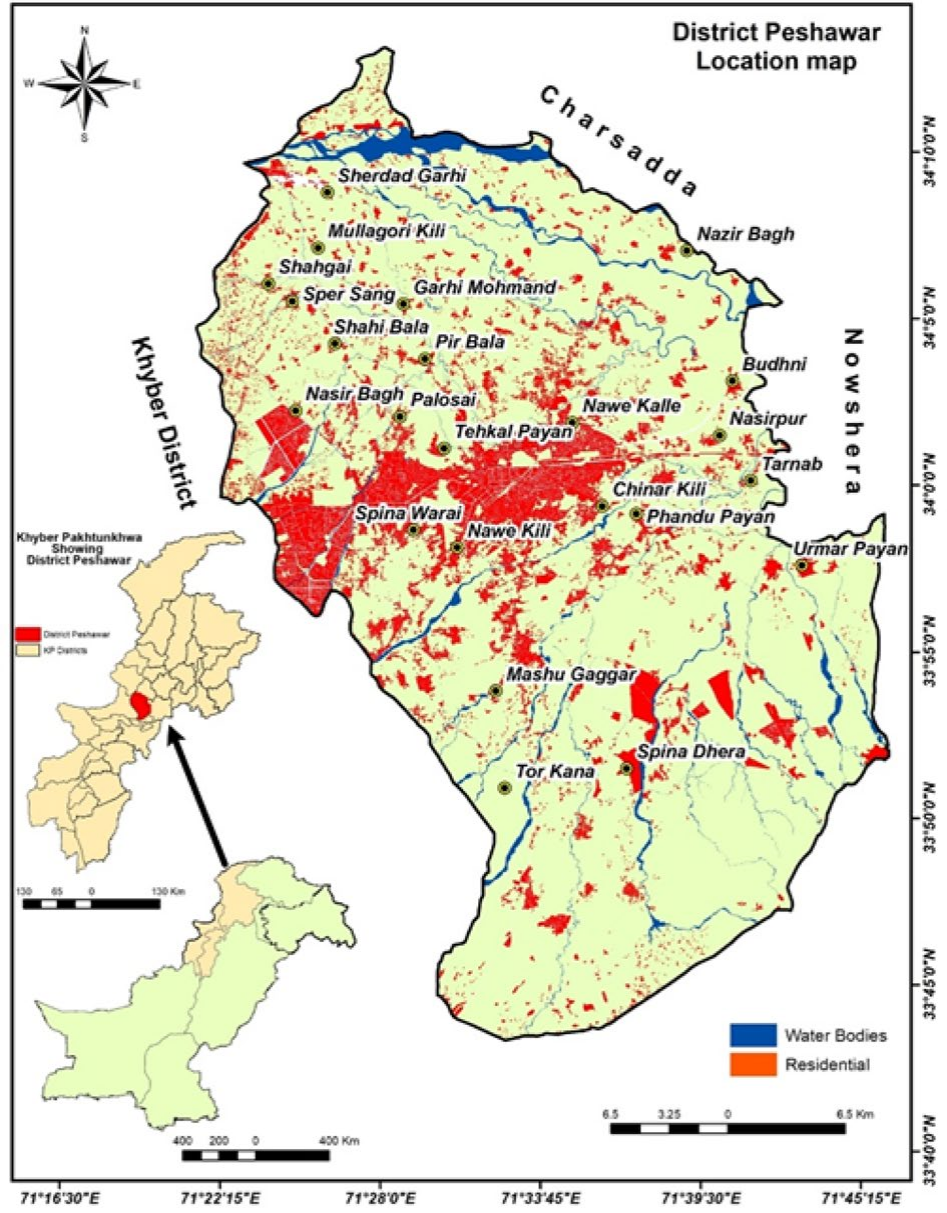
Study area map showing the built-up area (red color), major settlements and major stream (blue color). Source: Open street map (https://www.openstreetmap.org).

District Peshawar (33°.40′0.00″ to 34°.10′0.00″ N latitude and 71°.25′0.00″ to 71°.45′0.00″ E longitude), which is the capital of the Khyber Pakhtunkhwa (KP) province of Pakistan (Fig. 1) was selected for this research based on its exponential population growth over three decades due to the settlement of internally displaced people (IDP) and a massive influx of Afghan refugees since 1979^37,38^. This has created multifaced pressure on natural resources, noticeably the deterioration of groundwater quality and its impact on human health^25^. Peshawar is the ninth-largest city of Pakistan; mostly, the population is urban. Peshawar is a plain area, having a total area of 1257 km^2^. Its climate is scorching in summer and mild in winter. The Valley of Peshawar is covered with consolidated deposits of silt, sands, and gravel of recent geological times^39^. The mean maximum summer temperature exceeds 40 °C (104 °F) during the hottest month (July), and the mean minimum temperature is 25 °C (77 °F). The mean minimum temperature during the coldest month (December) is 4 °C (39 °F), while the maximum is 18.3 °C (64.9 °F), and the average annual rainfall in district Peshawar is 30.48 cm during monsoon^40^.

### Collecting groundwater samples and laboratory analysis

**Figure 2:**
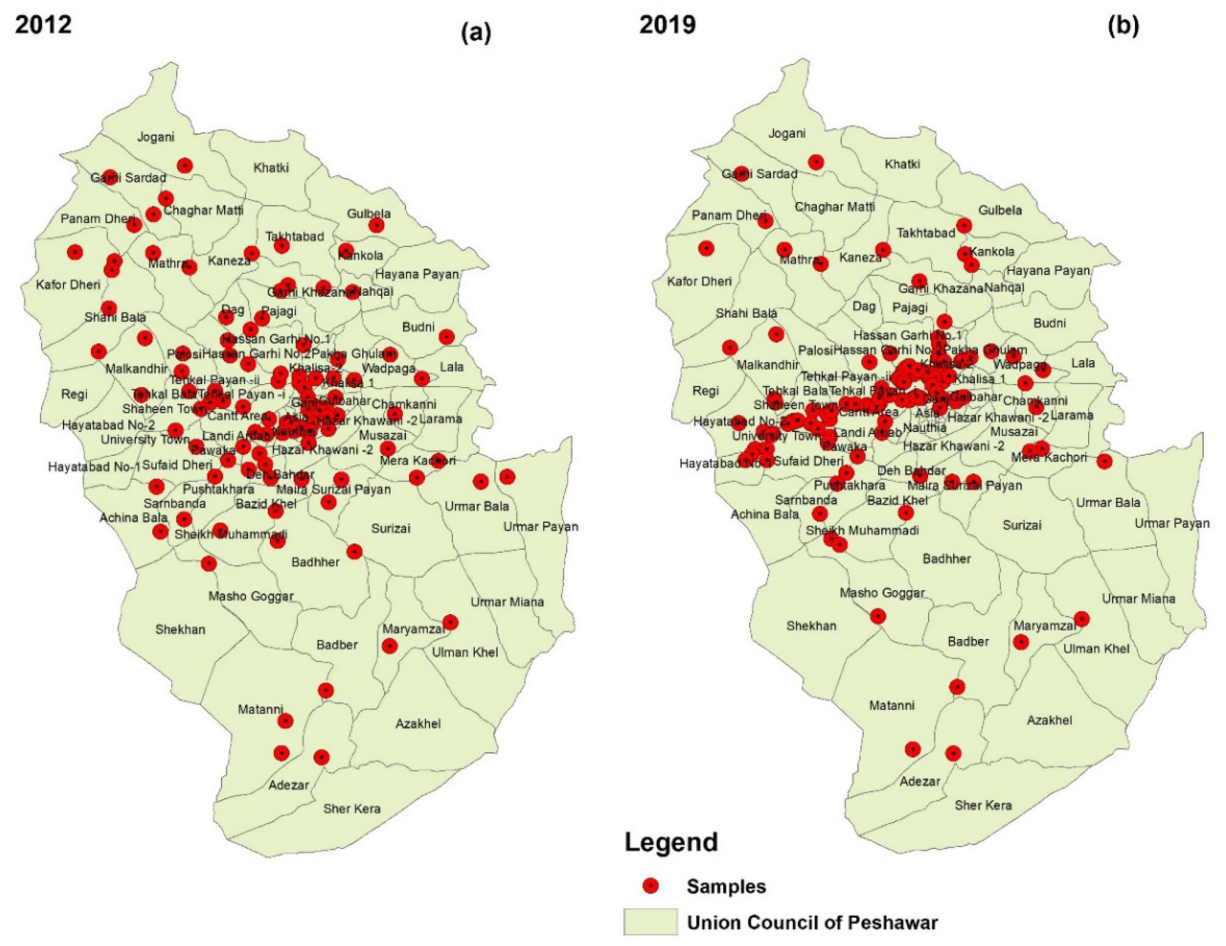
Showing the sample location of tube wells (**a**) the 2012 sample locations in different Union Councils and (**b**) the 2019 sample location point.

A total of 105 and 112 groundwater samples were collected in 2012 and 2019, respectively, from different locations of 93 union councils of Peshawar district (Fig. 2) for analysis of different water quality parameters (Table 1) and to assess the impact of land use/landcover change on groundwater quality. The groundwater samples were analyzed for seven water quality parameters (Table 1) using the standard analytical methods as given in the Standard methods of the American Public Health Association, American Water Works Association for water and wastewater (23rd Edition)^41^.

**Table 1:** Water quality parameters and methods of analysis (APHA, 23rd edition).

Parameters	Analysis method
pH	pH meter
Electrical conductivity (µS/cm)	E.C meter (Brand: Hanna)
Turbidity (NTU)	Method of 2130. B using turbidity meter
Calcium (mg/L)	EDTA titration, standard method 3500-Ca. B
Magnesium (mg/L)	EDTA titration, standard method 3500-Mg. B (2012)
Nitrate as nitrogen (mg/L)	Standard method 4500-NO_3_ B using spectrophotometer
Chloride (mg/L)	Standard method-4500 Cl-B

### Spatial analysis of water quality parameters

The inverse distance weighted (IDW) interpolation technique was used to generate Spatio-temporal surface maps for each water quality parameter in the ArcGIS 10.5^42^. The IDW is a commonly used algorithm for interpolating point data in spatial terms, allowing estimates of values other than the sample points measured. This assumes that each point of measurement has a local influence, which fades away at a distance, and the strongest influences are always near the point observed^43^. The IDW technique was preferred over other interpolation techniques because the unsampled locations' predicted values are within the maximum and minimum values of the observed data.

### Land use/land cover changes

To quantify (2012–2019) land use/land cover changes, remotely sensed LANDSAT 7 (30 m spatial resolution) and LANDSAT 8 satellite data were used (downloaded from the USGS website http://www.earthexplorere.com). The 30-m resolution image was acquired on 15 May 2012, and another image was obtained from 18 May 2019, respectively. Both images were subjected to a series of pre-processing techniques, including geometric and radiometric corrections using the Arc GIS 10.5. Both images were spatially referenced in the Universal Transverse Mercator (UTM) projection system (zone 42 north) with the World Geodetic System (WGS) 1984 as a datum. For the land-use classification, both images were processed separately and were subjected to supervised maximum likelihood classification using training sample polygons. The classification of the maximum probability assumes that statistics are normally distributed for each class on each strip and calculates the probability that a pixel belongs to a specific class. All pixels are classified unless, choose a probability threshold. The highest probability is assigned to each pixel (that is, the maximum likelihood). Based on the author's field experiences, a total of four major land use classes were present, including (1) agriculture (2) barren land (3) built-up areas, and (4) water bodies. After that, zonal statistical tools were applied to land use/land cover and all water quality parameters to evaluate the land use/landcover effect on water quality. It is a statistical tool that calculates each zone values based on the values from another dataset. In this study, the centroid of the union councils was converted into a point feature. With the help of these points feature, the water quality values were obtained from the interpolated rasters and land-use/landcover values in the form of tables using the “zonal statistics as table” tool in ArcGIS 10.5.

### Health and water contaminated sources data for the management policy

Human health is more affected by bacteria, pathogens, organic substances, and various minerals present in drinking water. In developing countries, there is a significant proportion of people's health issues due to unsafe water microbial contamination, about five million children's deaths occur to the contamination in drinking water^44^. This situation is increasing day by day due to the rapid population growth, which ultimately results in poor water quality management. It is estimated that in Pakistan, 30% of all diseases and 40% of all deaths are due to unsafe drinking water^45^.

To analyze the water-related disease in the study area, major diseases (methemoglobinemia, Urinary Tract Infections, Peptic Ulcer Diseases, Dental Caries) District wise data were collected from the Health Department Of Khyber Pakhtunkhwa, which is freely available for public for research and decision making purposes. The data was only 4 months from January to April of the 2 years (2012 and 2019). All guidelines and standard operating procedures were followed to access clinical data from district health office, Peshawar. The tube wells and the stream discharge data were obtained from the Water and sanitation services Peshawar (WSSP) to properly plan and sustain the groundwater.

## Result and discussion

### Analysis of groundwater quality parameters

**Figure 3:**
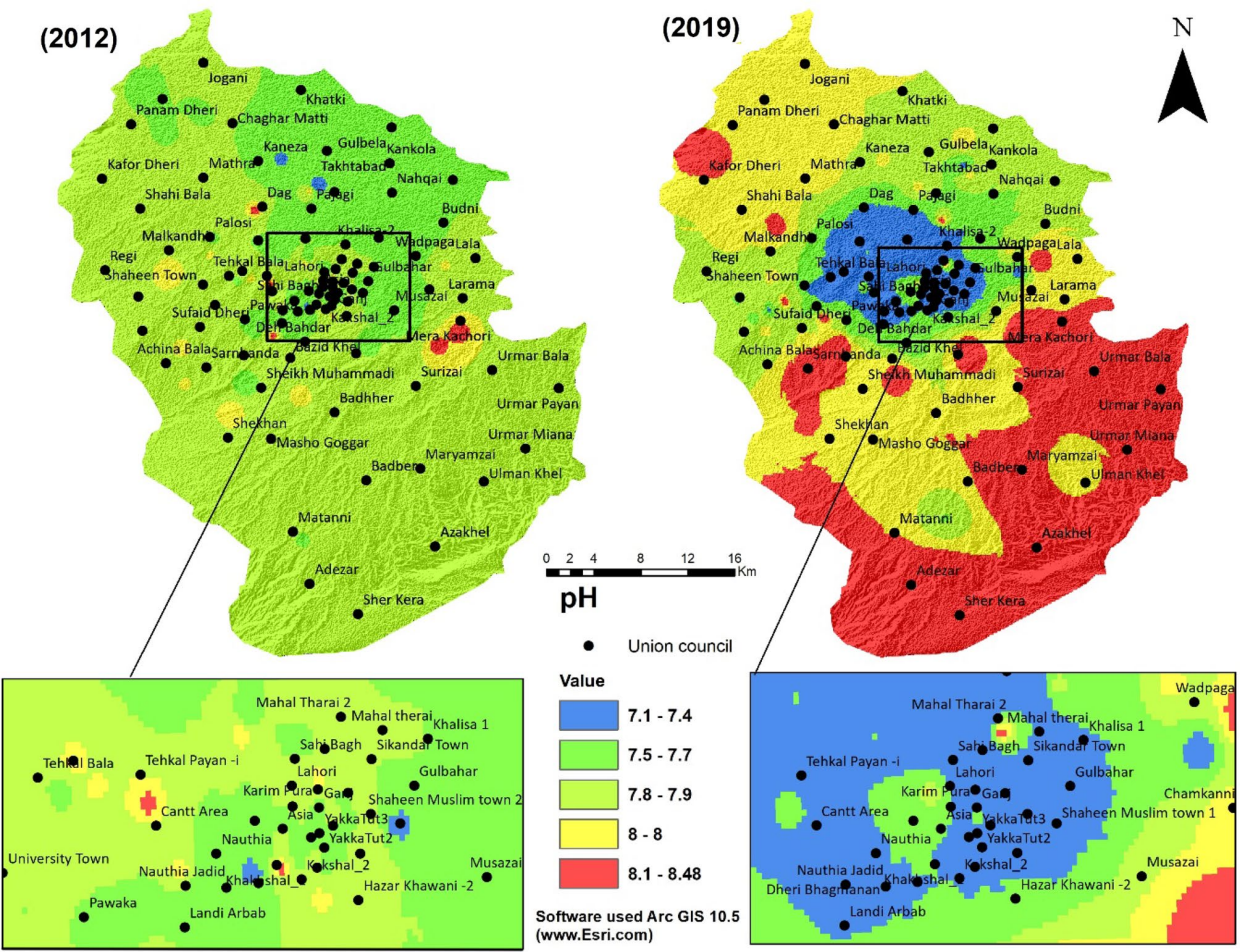
pH of groundwater of the 2 years (2012 and 2019).

The minimum and maximum values of pH for the year 2012 were found to be pH = 7.1 and pH = 8.08, and for 2019, the minimum and maximum values in the study area were pH = 7.02 and pH = 8.0. Many factors, including both natural and manmade, can affect the pH in water. However, our results show that the minimum and maximum pH values from 2012 to 2019 remained unchanged within the study area (Fig. 3), but there is a noticeable change over the years. Spatiotemporal interpolation of pH displays an increasing trend, especially in the Southeastern region of study area Union councils from Mera Kachori to Sher Kera and in the northwest from Jogani to Malakandair.

Spatial interpolation map of pH for the year 2012 shows that approximately in all districts pH values ranged between 7.5–8 except in Mera Kachori, but in 2019 pH value of these areas increased to 8–8.5 (Fig. 3).

Over the years, runoff generated from construction sites in urban areas and agriculture sites may have caused spatiotemporal groundwater quality parameter changes. From 2012 to 2019, the highest concentration of the pH was found in the built-up area. This could probably be due to the construction and the wastewater discharge from the domestic areas, while in the agriculture area, the highest pH concentration could be the use of pesticides and fertilization applications in the area. The pH values of all the samples were within the limit of WHO guidelines of water quality (Table 2).

**Table 2:** Groundwater quality parameters.

Parameter	2012			2019		
	Range	Average	STD	Range	Average	STD
Calcium (mg/L)	60.05–367.73	186.42	30.33	68.46–677.08	226.85	55.57
Chloride (mg/L)	13.30–94.79	31.1	9.84	10.02–87.98	32.62	11.78
Electric Conductivity (µS/cm)	389.82–1691.69	824.51	180.54	253.90–1695.57	679.46	182.2
Magnesium (mg/L)	65.55–429.24	178.5	52.7	10.7–373.95	165.6	74.74
Nitrate (mg/L)	0.09–41.72	19.07	7.67	1.25–113.00	81.4	23.61
pH	7.10–8.08	7.77	0.1	7.03–8.48	7.9	0.22
Turbidity (NTU)	0.16–7.09	0.62	0.33	0.03–21.87	0.09	0.8

*STD* standard deviation.

**Figure 4:**
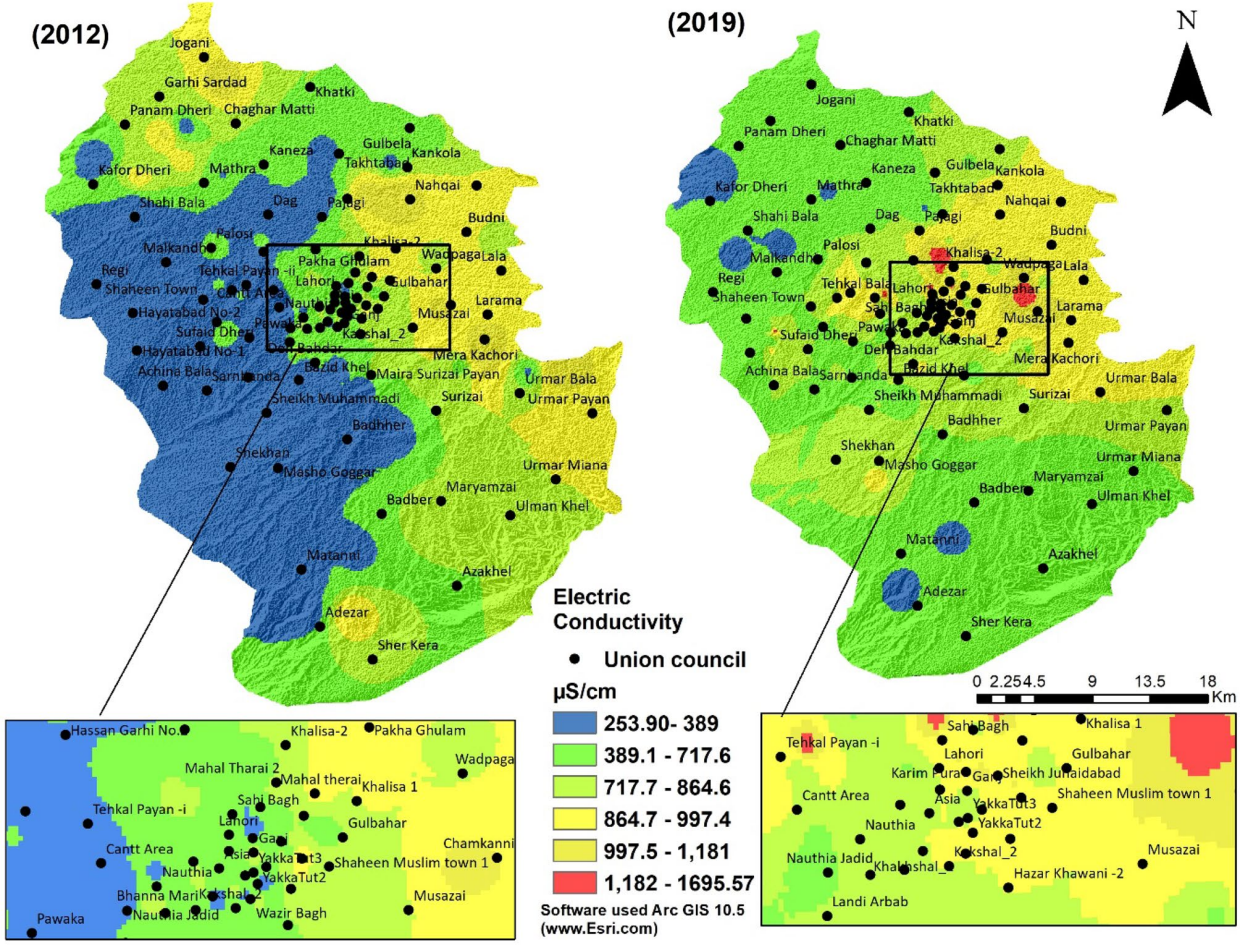
Electric conductivity of groundwater in the periods of 2012 and 2019.

Electrical conductivity is essential for the assessment of the water quality of a particular area. It acts as a baseline for measuring other water quality parameters as conductivity measures dissolved ionic concentration^46^. A significant variation in the conductivity value indicates a change in water quality or inclusion of some contaminants or indicates that the area is affected by some source of pollution^47^. In study results, the minimum and maximum value of EC recorded in 2012 was 389.82 µS/cm and 1691.69 µS/cm, while in 2019, the minimum and maximum EC values were 253.90 µS/cm and 1695.57 µS/cm, respectively (Fig. 4). The high value of EC in some areas such as Jogani, Mera Kochorai, and Umar Bala was may be due to the agricultural activity in these areas and leaching of minerals due to natural processes that occur around aquifer.

**Figure 5:**
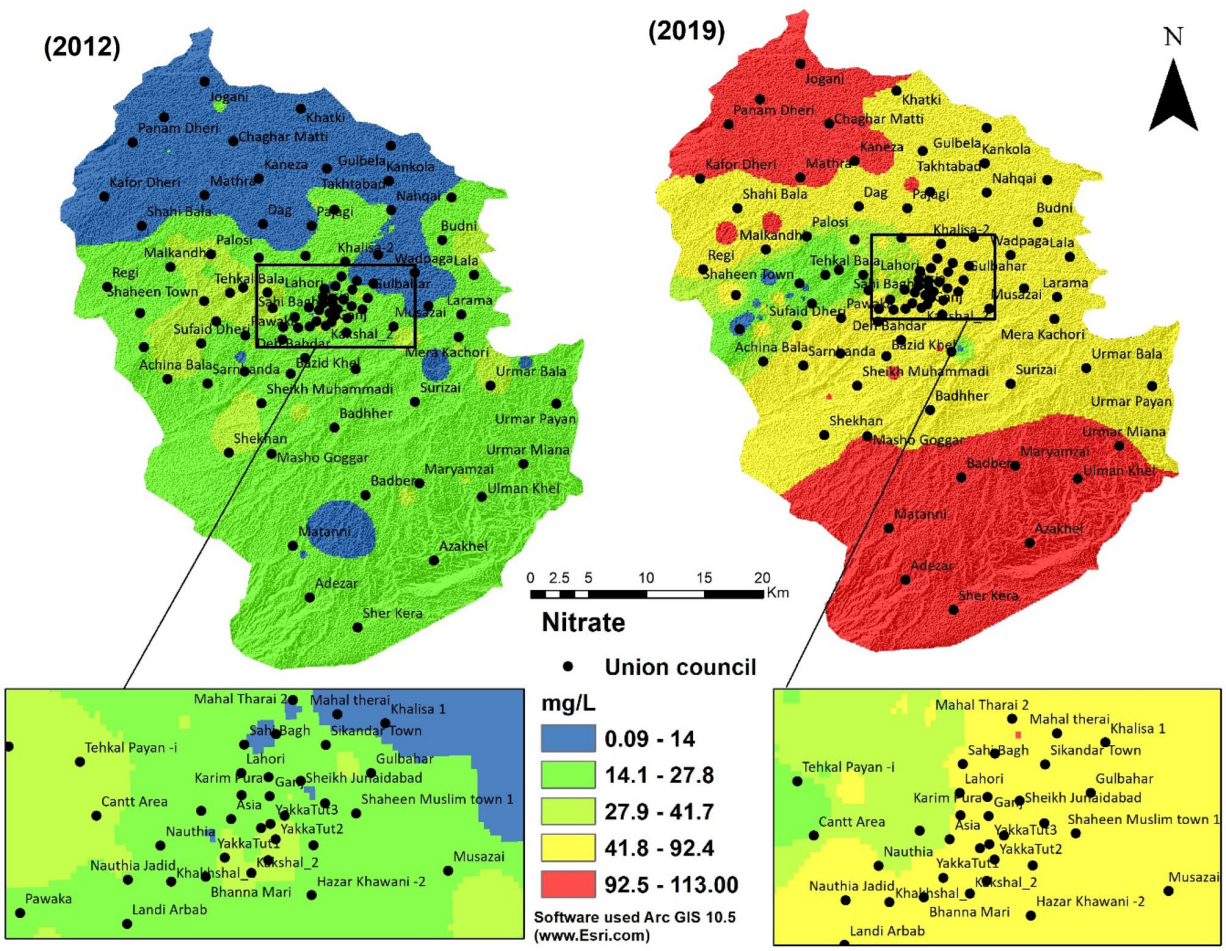
Nitrate of groundwater in the periods 2012 and 2019.

Nitrate mostly occurs in groundwater due to anthropogenic activities and exists in small amounts in surface water. Rainfall, lighting, industrial effluents, decomposition of organic compounds, and fertilizers are the major nitrate sources on the earth's surface^48,49^, which leach into groundwater and contaminate aquifers. High levels of nitrate can create enteric infections^50^. Though the adults may tolerate a high nitrate level with little or no expected adverse health issues, care must be taken while consuming water with nitrate concentration significantly more than 50 mg/L. The minimum value of nitrate in water quality results of 2012 was found to be 0.09 mg/L, and the maximum value was 41.72 mg/L while in 2019 the minimum value of the nitrate was 1.25 and the maximum was 113.00. The values of Nitrate in 2012 were within the limit of the WHO guidelines but in 2019, the values were much higher than the recommended limit (Fig. 5).

Spatial analysis of 2012 water quality shows that nitrate values were within WHO guideline value despite agricultural activities. Over the years, with an increase in urban sprawl in the center of the city like in Hayatabad, Malakandair, and Tehkal Bala, sewage water is leaching into the ground and mixing in water supplies in 2019. The nitrate values are higher in southeast peripheral areas due to rapid urbanization and increased agricultural activities. Farmers use pesticides and large amounts of fertilizers to produce a high yield from small agricultural fields^35^. These areas have intensive agricultural activities. Thus the increase of nitrate from 2012 to 2019 is substantial. Babies consume large quantities of water relative to solid food, mainly if the water is used to mix powdered or concentrated formulas or juices. Therefore they are at high risk by consuming contaminated water. Infants have immature digestive systems, which allow the reduction of nitrate to nitrite^51^. In particular, the presence of nitrite in newborns' digestive tract can lead to a disease called methemoglobinemia. According to the Khyber-Pakhtunkhwa Health Department, this disease increases in infants born in Peshawar's district (study area). The following Table 3 shows the treatment of newly born infants who had methemoglobinemia. In 2012, 46% of the newborn children were treated for methemoglobinemia while in 2019, it increased to 62% (Table 3), indicating that it will be the disaster in the future. A high amount of nitrate in potable water can also degrade the quality of water by increasing algal growth.

**Table 3:** Patients' treatment data from January to April in 2012 and 2019.

2012				2019			
Population	Child born	Treatment for methemoglobinemia	Percentage	Population	Child born	Treatment for methemoglobinemia	Percentage
3,219,000	27,361	12,717	59	4,269,079	36,287	22,439	72

Source: Health Department of Khyber Pakhtunkhwa.

**Figure 6:**
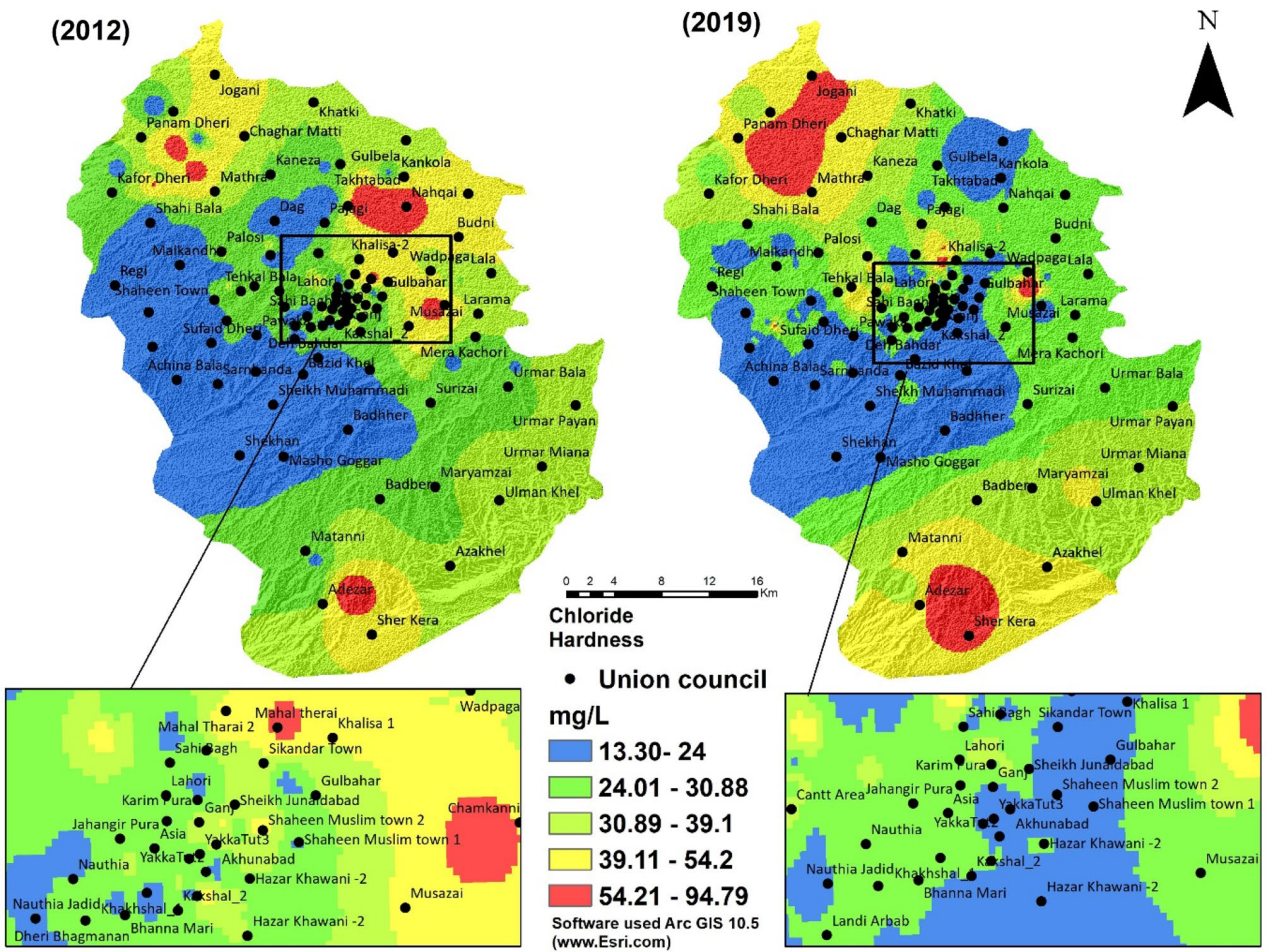
Chloride hardness of groundwater of 2 years (2012 and 2019).

The chloride content level in fresh and uncontaminated water should be less than 250 mg/L^52^. According to the laboratory analysis of samples taken from the study area, the minimum and maximum values of chloride in 2012 were 13.30 mg/L and 94.79 mg/L, while in 2019, the minimum and maximum values were 10.02 mg/L and 87.98 mg/L, respectively (Fig. 6).

Although Calcium is found in human teeth and bones as well as plays a vital role in blood clotting and vascular movements, however, the higher concentration of Calcium in the gastrointestinal tract of people having vitamin D- deficiency hyper calcimine and milk-alkali syndrome can reduce the absorption of other vital elements like iron and zinc^53^.

**Figure 7:**
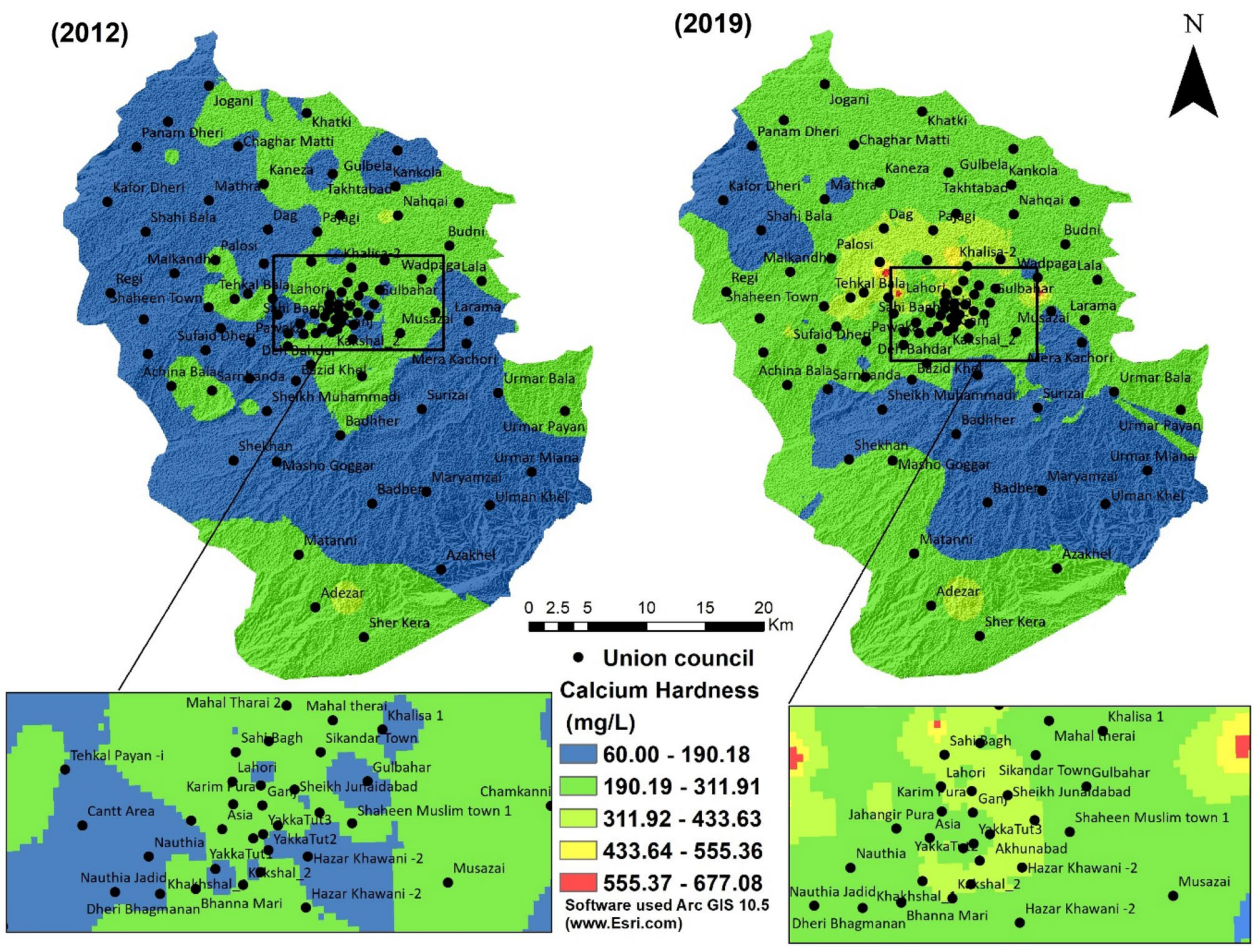
Calcium hardness of groundwater of the 2 years (2012 and 2019).

**Figure 8:**
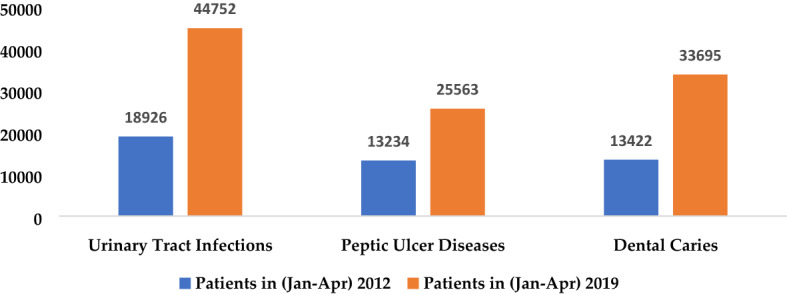
Water quality-related diseases in 2012 and 2019 in the Peshawar district. Source: Health Department of Khyber Pakhtunkhwa^55^.

Calcium is part of our dietary intake; therefore, on the basis of water hardness, according to drinking water quality guidelines of WHO 2017, an acceptable level of Calcium in drinking water is 100–300 mg/L. Water sample analysis results show that minimum and maximum concentration of Calcium in 2012 and 2019 were 60.05 and 367.73 mg/L, and 68.46 and 677.08, respectively (Table 2, Fig. 7). As calcium is directly related to water hardness, the availability of carbonate or fluoride contents in drinking water leads to CaF_2_ and CaCO_3_. These compounds can cause kidney tubular damage^54^. In the study area second most occurring disease is Urinary Tract Infections, which have 18,926 patients in 2012 (Jan–Apr), and in 2019 (Jan–Apr), the patients increased to 44,752 (Fig. 5). One of the reasons could be the occurrence of Calcium in the drinking water. The effect of acids on the teeth enamel surface causes dental caries. Dental caries patients in the study area were 13,422 in 2012 (Jan–Apr), and in 2019 (Jan–Apr), the number of patient was 33,695, which increased almost double (Fig. 8).

**Figure 9:**
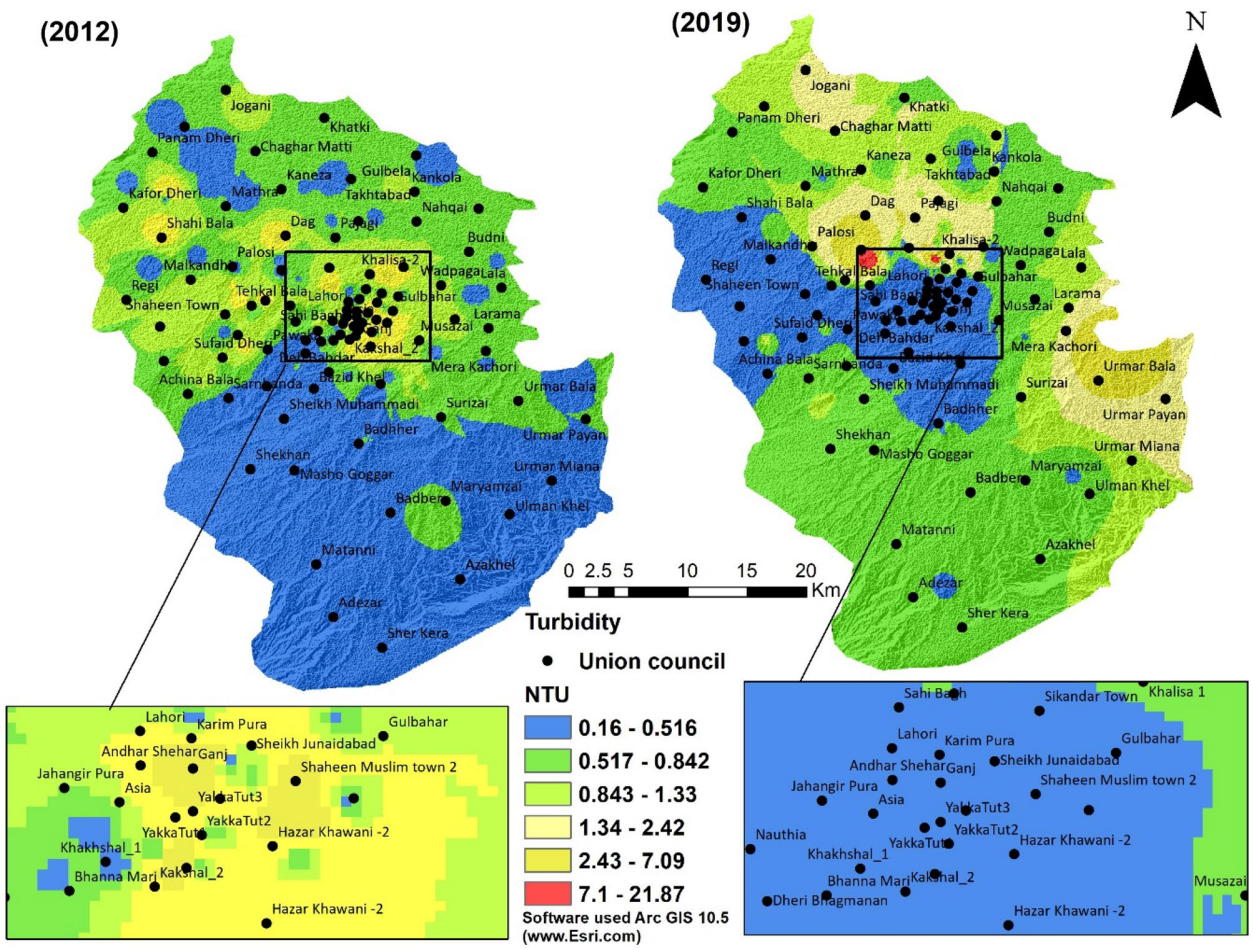
Turbidity of groundwater of the 2 years (2012 and 2019).

Turbidity, an aesthetic parameter, is used as a measure for the clarity of the water. It is caused due to different suspended materials such as silt, clay, and biological suspension in water. High turbidity levels affect light penetration in surface water bodies and reduce their ecological productivity^56^. In the study area, the minimum and maximum turbidity in 2012 was 0.16 NTU and 7.09 NTU, while in 2019, the minimum and maximum turbidity were 0.03 NTU and 21.87 NTU (Fig. 9).

A comparison of turbidity in Fig. 8 depicts a slight increase in water turbidity in recent years but still within the limit of WHO guidelines. One of the leading causes of increasing turbidity could be wastewater intrusion into the groundwater aquifers. City planners must draft a water policy to cope up with rapid urbanization. Designing and maintaining efficient drainage, wastewater disposal, and treatment systems are required for metropolitan cities to provide clean and affordable water to citizens. Public awareness programs may be conducted to improve drinking water supply and water sanitation and disposal facilities.

### Land use/landcover changes

**Figure 10:**
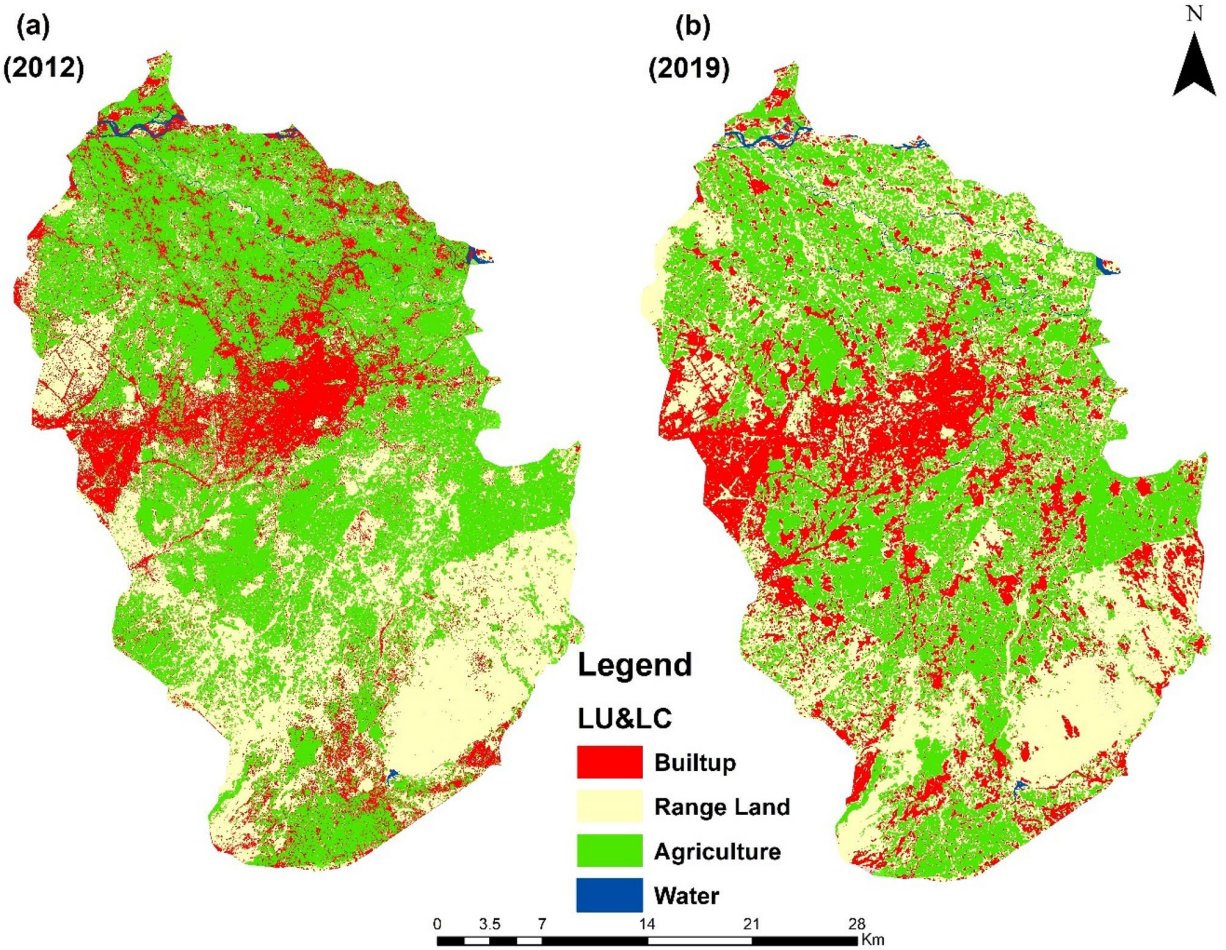
Landuse/landcover of the study area. (**a**) The 2012 land use/land cover. (**b**) The 2019 landuse/landcover.

The Maximum Likelihood (supervised) classification was performed for the two different years (2012, 2019) and the analysis of these classified images provided information about the land use/landcover features of the study area (Fig. 10). The analysis of the year 2012 classified image revealed that water bodies covered 7.163 km^2^ (0.56%) area, 441.173 km^2^ (34.86%) area by rangeland, 229.467 km^2^ (17.86%) area by Built-up and agricultural land cover was 606.865 km^2^ (47.24%) (Table 4). In 2019, the classification results showed that the water bodies covered area was 7.786 km^2^ (0.61%). The rangelands were 484.718 km^2^ (37.73%), 281.236 km^2^ (21.89%) area were built-up, and agriculture land cover were 510.928 km^2^ (39.77%) (Table 4). This analysis shows a decrease in the agricultural land whereas an increase in the rangeland, Water land and built-up has been noticed.

**Table 4:** Showing the landuse/landcover classes of district Peshawar.

2102			2019		
Land use/landcover	Area in km^2^	Percentage	Area in km^2^	Percentage	Landuse/landcover changes
Range land	441.173	34.34	484.718	37.73	43.545
Built-up	229.467	17.86	281.236	21.89	51.769
Agriculture	606.865	47.24	510.928	39.77	− 95.937
Water	7.163	0.56	7.786	0.61	0.623

### Effect of land-use/landcover changes on groundwater quality

The impact of land use/land cover on water quality depends on the type of land category. For example, in extensive agriculture, large farms are being cultivated with relatively lower inputs of pesticides, and fertilizers which have not much more impact on groundwater. While in intensive agriculture, small farms are cultivated wherein there is high-level use of fertilizers, and pesticides, which have more impacts on groundwater. Also, build-up land shows a wide variation in groundwater quality, which depends on the disposal of polluted water which is used for anthropogenic activities and contaminant places. The zonal statistic tool was used to estimate the association between different land classes and mean concentration changes for all water quality parameters. This suggests that the groundwater in the region having more influence from land use/land cover conditions. All the 2019 water quality parameters have more variation than the 2012 water quality parameters shown in Table 5.

**Table 5:** The effect of land-use/landcover on water quality parameters.

Parameters	2012				2019			
	Min	Max	Mean	STD	Min	Max	Mean	STD
**Agriculture**								
pH	7.10	8.06	7.77	0.11	7.04	8.48	7.92	0.21
Conductivity (µS/cm)	473.83	1633.30	832.98	198.71	258.12	1485.96	686.81	185.87
Turbidity (NTU)	0.19	3.00	0.64	0.31	0.04	21.87	0.99	0.83
Calcium (mg/L)	60.05	341.06	186.75	30.69	68.58	595.66	223.25	55.17
Chloride (mg/L)	13.34	90.87	31.06	10.77	10.02	87.41	32.99	12.17
Magnesium (mg/L)	65.55	415.63	179.75	55.88	11.55	373.95	175.68	68.81
Nitrate (mg/L)	0.09	41.08	18.50	7.86	8.92	113.00	84.59	19.54
**Rangeland**								
pH	7.17	8.07	7.78	0.09	7.10	8.46	7.91	0.25
Conductivity (µS/cm)	389.82	1664.89	841.66	157.62	255.88	1472.85	609.41	172.61
Turbidity (NTU)	0.19	3.30	0.55	0.27	0.05	15.16	0.66	0.40
Calcium (mg/L)	62.10	367.73	187.01	32.30	92.38	578.80	226.58	51.96
Chloride (mg/L)	13.30	92.98	32.48	8.87	10.27	84.43	35.81	10.49
Magnesium (mg/L)	85.42	428.48	181.22	49.01	11.31	312.75	173.29	82.54
Nitrate (mg/L)	0.16	41.46	18.28	7.51	3.93	112.96	85.82	28.71
Water								
pH	7.59	7.77	7.68	0.05	7.09	8.44	7.92	0.16
Conductivity (µS/cm)	825.87	1277.23	979.70	120.00	256.20	1280.07	672.66	148.02
Turbidity (NTU)	0.46	1.04	0.71	0.13	0.06	9.33	1.01	0.58
Calcium (mg/L)	171.02	217.11	193.29	11.58	105.29	459.24	226.70	42.71
Chloride (mg/L)	27.16	46.95	38.93	5.27	10.63	76.65	35.84	12.14
Magnesium (mg/L)	189.42	316.23	233.85	36.99	16.32	364.32	200.70	78.65
Nitrate (mg/L)	5.14	19.86	9.83	3.64	9.68	112.50	88.24	18.74
**Built-up**								
pH	7.16	8.08	7.78	0.10	7.03	8.47	7.79	0.27
Conductivity (µS/cm)	394.33	1691.69	744.67	171.23	253.90	1695.57	705.12	176.66
Turbidity (NTU)	0.16	7.09	0.77	0.46	0.03	16.93	0.70	0.88
Calcium (mg/L)	75.67	354.55	181.46	25.45	68.46	677.08	241.60	62.94
Chloride (mg/L)	13.71	94.79	26.83	9.13	10.22	87.98	28.26	9.79
Magnesium (mg/L)	82.15	429.24	165.00	52.43	10.87	373.16	114.14	72.08
Nitrate (mg/L)	0.27	41.72	22.65	6.74	1.25	112.38	64.91	27.69

Groundwater quality parameters show an increasing trend for the year 2019 (Table 5) in all land-use types (agriculture, rangeland, water, built-up) compared to the year 2012. In agricultural areas concentration of nitrate and magnesium switches (19.54 mg/L with a Standard deviation of 68.81 mg/L). This means that the amount concentration of these parameters was significantly increased in the year 2019 compared to 2012. The reason for a high concentration of nitrate, magnesium, and calcium could be the frequent use of pesticides and fertilizers at the farm level. In rangeland, the concentration of nitrate, calcium, and magnesium increases by 10.49 (mg/L), 82 (mg/L), and 28.71 (mg/L), respectively, due to the high infiltration of chemicals by nearby industries. Surface water is contaminated and polluted by mixing waste or drainage water from the household, enlarging Calcium and magnesium's concentration by 18.74 (mg/L) and 82.54 (mg/L) standard derivation, which is alarming for residents. The development in infrastructure amount of Calcium is increased in 2019 compared to 2012 (241.60 (mg/L) and 181.46 (mg/L), respectively), as shown in Table 5. Calcium's high level in built-up areas is due to the decomposing of chemicals and waste materials from nearby solid waste damping zones.

### Management strategies

Management strategies should include pollution prevention at source^57^, upgrading drinking water distribution lines, monitoring and maintaining them properly, and creating public awareness of using safe and clean water. The main reason in the study area is that there is no proper usage of dumping sites. The residence put all the garbage directly into the stream; also, the toilet pipes are come out directly to the streams, which are the leading causes of water pollution. Many tube wells extraction points were situated near the streams and canals by which household drainage and sewage systems opened. In the study area, strict compliance with national quality standards is required to curb pollution at the source. According to the PCSIR laboratory, Peshawar's water quality is poor and unsuitable for drinking without treatment Except Hayatabad^58^. The water supply and management departments and the Environmental Protection Agency (EPA) need to protect water resources from pollution^59^. There is a well-developed network of channels for irrigation purposes in the Peshawar district, but nowadays, due to anthropogenic activities, the water of these channels is very polluted. The residents also put their household wastes into these channels, causing water-related diseases (Tables 2 and 3). Irrigation water channels play a vital role in groundwater recharge in the adjacent areas; therefore, local administration needs to protect these irrigation channels from contamination and make water channel protection policy for future^60^.

In Pakistan, few industries have wastewater treatment plants to treat their effluent. The government should take strict action for its industrial effluent disposal, according to the National Environmental Quality Standards (NEQS) under the 1997 Act^61^. Public awareness campaigns should be started at schools, colleges, universities, and the community level to address the significance of safe drinking water. Rural communities should adopt water management practices, including rainwater harvesting, to reduce their water wastage. Local pieces of training should be conducted for improved water storage in houses and simple disinfection technologies of drinking water^62^. Many studies show that boiled drinking water reduces the risk of waterborne diseases^63,64^. Socioeconomic conditions, including education, household income, and living style, are correlated to the family's understanding of the importance of the quality of water they intake^65,66^. Thus water quality is directly related to the health and well-being of people^61,67^.

## Conclusions

This study concludes that the groundwater quality, which is the only major source of drinking in the study area (district Peshawar), has decreased in 2019 compared to 2012. Some major factors for this groundwater quality deterioration are urbanization, domestic wastewater discharge, and rising water demand for agriculture and domestic and industrial purposes. As a result, a sharp decline in the drinking water in terms of quality has been observed. More precisely, pH, Conductivity, Calcium, Chloride, Magnesium concentration in groundwater increased mainly near the built-up areas because of wastewater discharge, buildings construction, and other anthropogenic activities like water extraction, industrial activities. Nitrate concentration increased near the agricultural lands because of the excessive utilization of pesticides and fertilizer. As a consequence, water-related diseases were higher in 2019 as compared to 2012.

The introduction of precision agriculture enhances the efficiency of yield production by using a sufficient amount of water. Competent authorities should consider these results and formulates water usage policies and regulations. In the future, different models of neural networks would be used to analyze and monitor the factor or parameters affecting groundwater and surface water pollution. The study is set out to highlight the current groundwater scenario of Peshawar for sustainable water resources management.
